# Multimorbidity Analysis of 13 Systemic Diseases in Northeast China

**DOI:** 10.3390/ijerph17061817

**Published:** 2020-03-11

**Authors:** Jianxing Yu, Fangying Song, Yingying Li, Zhou Zheng, Huanhuan Jia, Yuzhe Sun, Lina Jin, Xihe Yu

**Affiliations:** 1 Social Medicine and Health Service Management, School of Public Health, No. 1163 Xinmin Street, Jilin University, Changchun 130021, Jilin, China; yjxjlu@163.com (J.Y.); a15703410189@126.com (F.S.); jdgwlyy@163.com (Y.L.); zhengzhou19@mails.jlu.edu.cn (Z.Z.); jhh_1994@163.com (H.J.); yuzhe35789@163.com (Y.S.); 2 Epidemiology and Biostatistics, School of Public Health, No. 1163 Xinmin Street, Jilin University, Changchun 130021, Jilin, China; jinln@jlu.edu.cn

**Keywords:** multimorbidity, systemic diseases, weighted networks

## Abstract

Background: Multimorbidity not only affects the quality of patients’ lives, but can also bring a heavy economic burden to individuals, families and society. The purpose of this study was to reveal the connections between diseases, especially the important role each disease played in the entire multimorbidity network. Methods: A total of 1,155,734 inpatients were enrolled through multistage stratified random sampling in Jilin Province in 2017. Categorical variables were compared using the Rao–Scott-χ2 test. Weighted networks were adopted to present the complex relationships of multimorbidity. Results: The distributions of the number of diseases differed significantly by gender, age and health insurance scheme (*P* < 0.001). Diseases of the respiratory system had the highest weight in multimorbidity in young people. Endocrine, nutritional and metabolic diseases and circulatory system diseases were often associated with other systemic diseases in middle aged and old people. Conclusions: Multimorbidity with respiratory system diseases in young people should not be overlooked. Additionally, effective prevention efforts that target endocrine, nutritional and metabolic diseases and circulatory system diseases are needed in middle aged and old people.

## 1. Introduction

Multimorbidity is defined as the presence of more than one health condition in an individual [1,2,3,4]. A person with numerous long-term health conditions has an increased risk of depression, hospitalizations, polypharmacy, loss of physical functioning, and even premature death [5,6]. In addition, multimorbidity places a substantial economic burden on the healthcare system [7,8,9,10], especially in the world’s most populous country, China [11,12], because patients with multimorbidity often require additional intensive treatment and monitoring by nurses, physicians and other healthcare staff [13,14]. Therefore, the identification of the key determinants of multimorbidity is an important public health aim that should be addressed urgently.

To date, most studies have focused on single diseases while ignoring multimorbidity [15,16,17]. Some studies on the prevalence of multimorbidity did not consider the significance of the connection between diseases [18,19]. According to a literature review, most of the recent studies were based on self-reporting of prevalence by specific populations in specific environments, and the sample size was not large enough. For example, Alessandra’s study [2] involved only 1099 elderly participants in the Kungsholmen Project, and they explored the role of age, gender, and socioeconomic status in the occurrence of chronic diseases and multimorbidity.

To the best of our knowledge, few studies have defined diseases based on the international standard (e.g., the International Statistical Classification of Diseases and Related Health Problems 10th Revision (ICD-10)). The ICD can be used not only to increase the accuracy of disease differentiation but also to monitor the incidence and prevalence of diseases and other health problems, providing a picture of the general health situation of populations. To facilitate a visual understanding of 13 systemic disease combinations, this study created a multimorbidity network graph.

## 2. Methods

### 2.1. Study Population

A large-scale cross-sectional survey was implemented among people who were hospitalized in 20 general hospitals in Jilin Province in 2017. A total sample size of 1,300,683 inpatients was obtained through multistage stratified random sampling. The measured variables were socio-demographics (such as age, gender and health insurance scheme), disease names and ICD-10 classifications. In this study, only 13 systemic diseases were included, and the inpatients with some diseases and conditions (such as certain infectious or parasitic diseases, pregnancy, childbirth and puerperium) were excluded because these diseases were infectious diseases, congenital diseases or other conditions (see the details in Part 1 of the online Supplementary Materials). In the end, this study explored multimorbidity in 1,155,734 inpatients.

### 2.2. Ethics Statement

The hospitals in the study informed the participants in advance that their disease information would be used for research, but participant names and any other personalized information were not collected. All research methods followed the guidelines of investigation, and written informed consent was obtained from all of the participants before data collection. All participants consented to this study, including the parents/legal guardians of those under the age of 18. This study was approved by the ethics committee of the School of Public Health, Jilin University. All procedures involving human participants complied with the ethical standards of the institutional and national research committees.

### 2.3. Data Collection

The target sample was obtained through multistage stratified random sampling. First, considering comprehensive analyses of geographical locations, economic development levels and the health service statuses of each region, Changchun, Jilin City, Tonghua, Baicheng and Yanbian were selected for inclusion. Then, one district and two counties in each administrative region were selected. Finally, on the basis of the administrative region, a total of 20 general hospitals were selected as the monitoring institutions in this study.

We included all consecutive inpatients, and the dataset consisted of data regarding age, gender, disease name, ICD-10 classification, and health insurance scheme from medical records. To improve the accuracy of the data, we recruited a professional team to organize the data. A total of 20 interviewers with medical backgrounds and proficiency in EXCEL were recruited and uniformly trained to check basic information (such as age, gender, and health insurance). In addition, a panel of 30 general practitioners with more than 3 years of work experience verified that the disease names matched the ICD-10 classifications.

The survey was implemented by the School of Public Health, Jilin University in Jilin Province in 2017. According to relevant regulations, we are sorry that the data cannot be shared.

### 2.4. Statistical Analyses

Categorical variables were expressed as counts or percentages and compared using the Rao–Scott-*χ*^2^ test. Weighted networks were adopted to present the complex relationships of multimorbidity. The nodes of the network represented the systemic diseases, and the size of each systemic disease node was the weight as it occurred together with all other diseases. The thickness of each line connecting two systemic disease nodes was the degree of association between the two systemic diseases. Degree was the number of nodes that a focal node was connected to, which measured the involvement of the node in the network. Network density and average degree were used to evaluate the sparsity of a network. The network density of an undirected graph with N nodes and M edges was defined as 2 M/N(N − 1), which described the portion of the potential connections (N(N − 1)/2) in a network that were actual connections (M). The average degree was defined as the average of the degrees of all nodes. The larger the network density (or average degree), the denser the network [20,21]. All statistical analyses were performed with R version 3.6.1 (University of Auckland, Oakland, New Zealand). Statistical significance was set at a *P* value < 0.05.

## 3. Results

We analyzed data from 1,155,734 patients from 20 general hospitals in Jilin Province. The subjects were divided into three groups according to the number of diseases (see details in Table 1). The distribution of the number of diseases differed significantly by gender, age and health insurance scheme (*P* < 0.001). In addition, the number of male patients was higher than that of female patients, and the proportion of male patients with two or more diseases was higher than the proportion of female patients with two or more diseases. Overall, the proportion of patients suffering from one disease decreased with age, while the proportion of patients suffering from three or more diseases increased. In terms of health insurance schemes, the proportion of patients with one disease with New-Type Rural Cooperative Medical System (NRCMS) and Urban Social Pension Scheme (USPS) insurance was higher than that of patients with one disease with Urban Employment Basic Medical Insurance (UEBMI), Urban Resident Basic Medical Insurance (URBMI) and other insurance, but the trend in patients with two or more diseases was the opposite.

**Figure 1:**
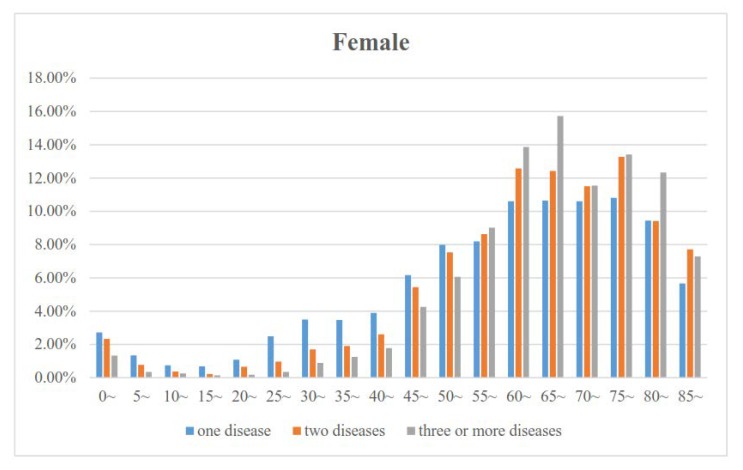
Association between age and the proportion of the number of diseases for females.

**Figure 2:**
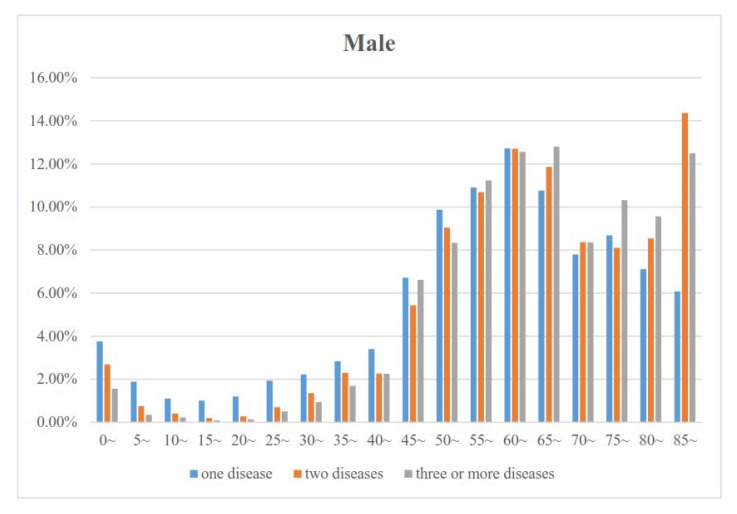
Association between age and the proportion of the number of diseases for male.

Figure 1 and Figure 2 show the proportions of the numbers of diseases by age for males and females. The proportion of the number of diseases decreased in those 0 to 19 years old, increased in those 20 to 64 years old, and then decreased slowly in those older than 65 years in both males and females. In addition, the proportion of three or more diseases was the lowest and the proportion of one disease was the highest in those less than 55 years old. However, the proportion of three or more diseases was the highest in those 55 to 84 years old. Figure 1 and Figure 2 also show that the age group with the lowest proportion and the age group with the highest proportion were the 10~19 and 60~69 years age groups, respectively.

**Figure 3:**
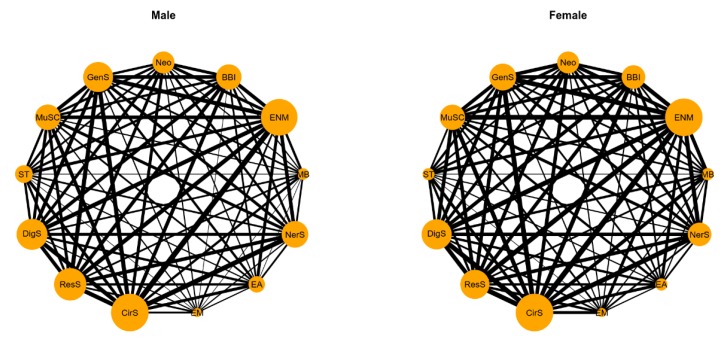
Multimorbidity networks by gender for 13 systemic diseases.

Table 2 shows the abbreviation of the 13 systemic diseases. The proportions of the ten most common two-disease combinations in the study population are presented in Table 3. In particular, endocrine, nutritional and metabolic diseases (ENM) and diseases of the circulatory system (CirS) had a relatively high proportion of combinations. Figure 3 shows a visual understanding of all the disease combinations. Endocrine, nutritional and metabolic diseases (ENM) and diseases of the circulatory system (CirS) had notably high weights in both males and females (illustrated by the sizes of the nodes) and frequently occurred together with other diseases (illustrated by the thickness of the lines connected to these nodes).

**Figure 4:**
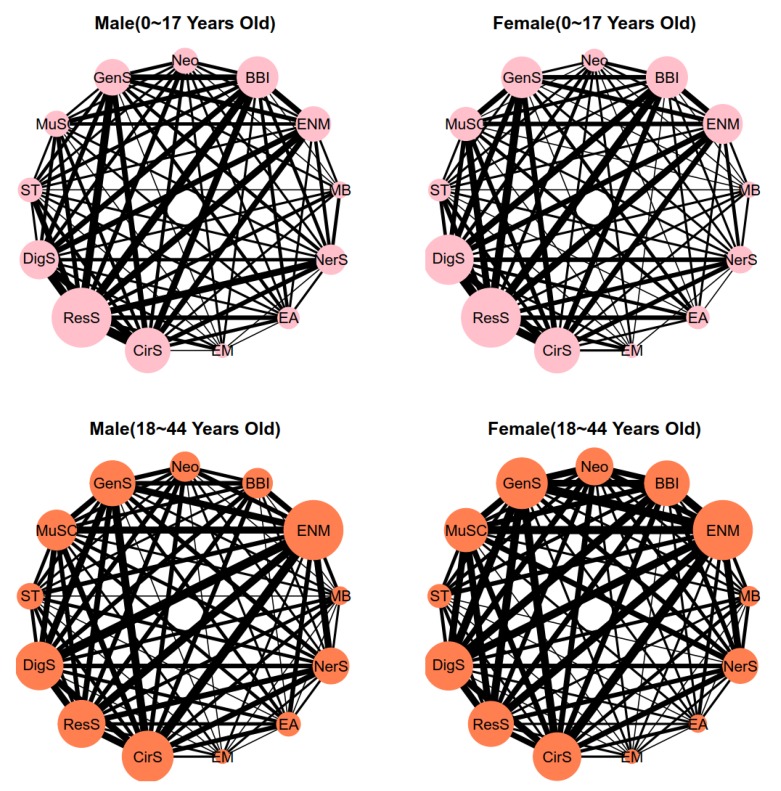

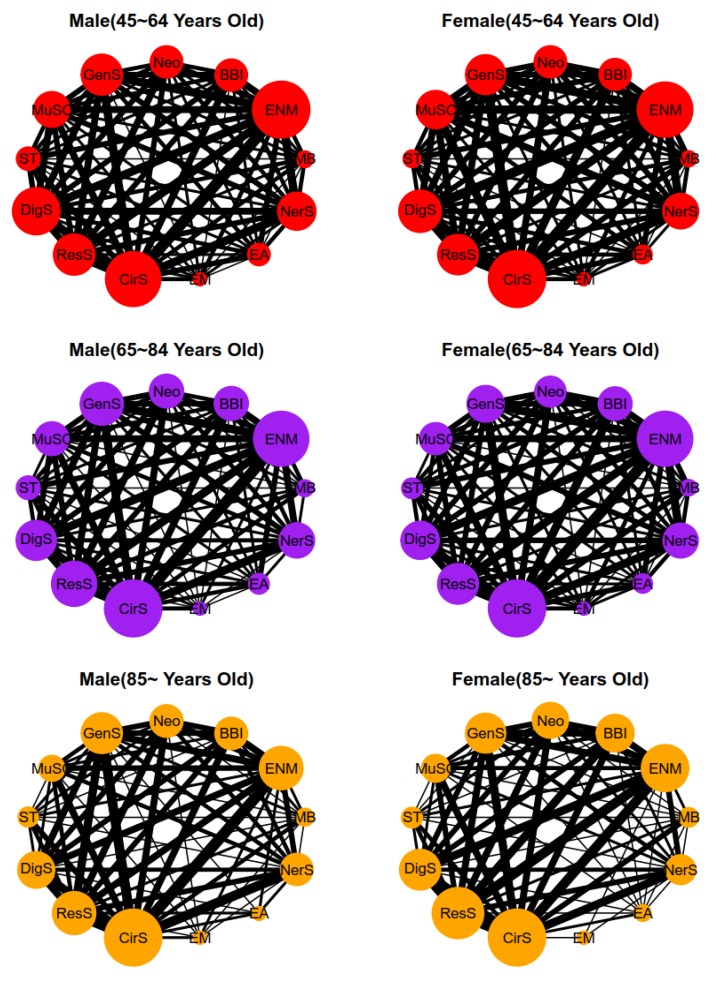
Multimorbidity networks by age group for 13 systemic diseases.

Figure 4 shows the multimorbidity networks by age for 13 systemic diseases. Generally, the multimorbidity networks were different for different age groups. In the age group 0~17 years, respiratory system diseases (ResS) had the highest weight. In addition, the “ResS-CirS”, “ResS-DigS”, and “ResS-BBI” had notably high connections in the multimorbidity networks, and the multimorbidity networks were similar among genders. However, endocrine, nutritional and metabolic diseases (ENM) had the highest weight, and the “ENM-CirS-ResS”, “ENM-CirS-DigS”, and “ENM-CirS-GenS” triangles had notably high connections in the multimorbidity networks in the age group 18~44 years. In addition, the size of Neoplasms (Neo) and blood and blood-forming organs diseases and certain disorders involving the immune system (BBI) nodes in male patients was bigger than that in female patients. In the age groups 45~64 years and 65~84 years, endocrine, nutritional and metabolic diseases (ENM) and circulatory system diseases (CirS) had the highest weights. Similar to the age group 18 to 44 years, the “ENM–CirS–ResS”, “ENM–CirS–DigS”, and “ENM–CirS–GenS” triangles had notably high connections in the multimorbidity networks. Notably, the average degrees of the connections/triangles in the age groups 45~64 years and 65~84 years were much higher than those in the age groups 0~17 years and 18~44 years. Finally, circulatory system diseases (CirS) had the highest weight in the group aged 85~ years old and above, but the “ENM-CirS-ResS”, “ENM-CirS-DigS”, and “ENM-CirS-GenS” triangles still had notably high connections in the multimorbidity networks (for the proportions of the ten most common two-disease combinations in different age groups, see online Supplementary Materials Tables S2 and S3).

**Figure 5:**
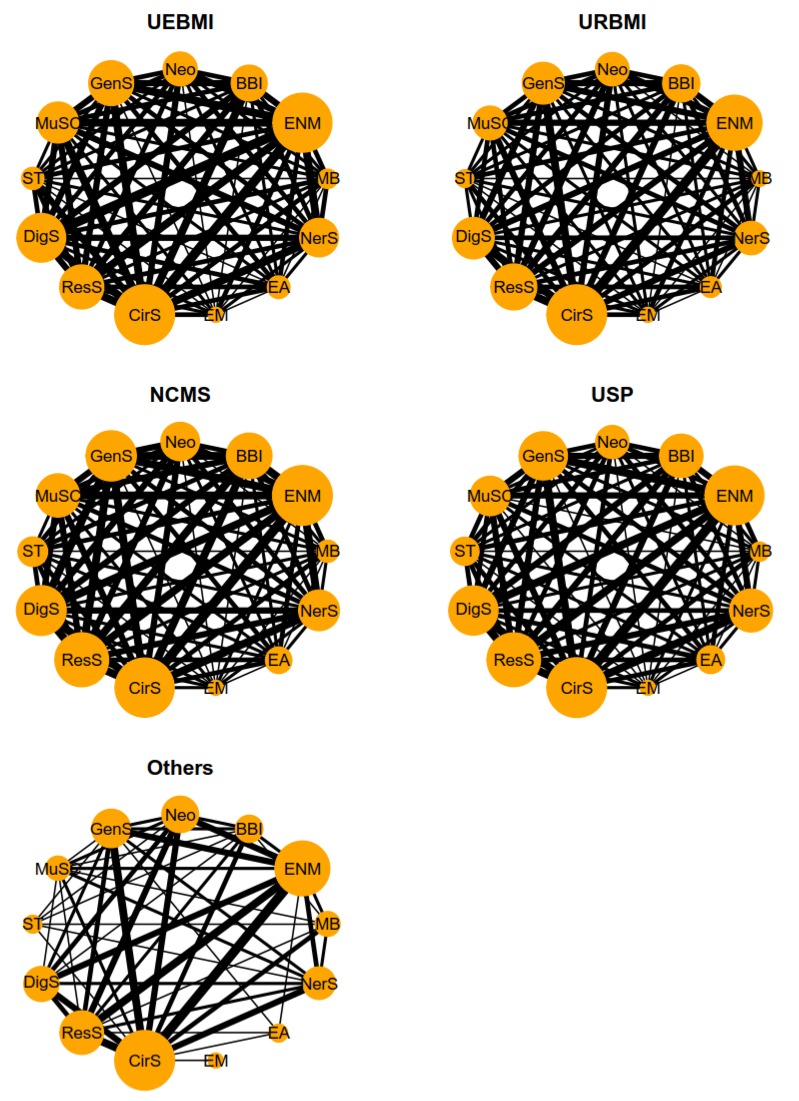
Multimorbidity networks by health insurance schemes for 13 systemic diseases.

The multimorbidity networks were similar in the four kinds of health insurance schemes (except others), and ENM (endocrine, nutritional and metabolic diseases) and CirS (diseases of the circulatory system) had a relatively high proportion of combinations. Figure 5 shows a visual understanding of all the disease combinations. ENM (endocrine, nutritional and metabolic diseases) and CirS (diseases of the circulatory system) had notably high weights in UEBMI, URBMI and NRCMS and frequently occurred together with other diseases (for the proportions of the ten most common two-disease combinations in different age groups see online Supplementary Materials Table S4).

## 4. Discussion

Multimorbidity is believed to bring great challenges to, and have important impacts on, current public health. Employing large cross-sectional data from a survey of Jilin Province, this study explored multimorbidity in hospitalized patients with regard to 13 systemic diseases, and examined the association of combinations comprising two diseases from these 13 systemic diseases. If we are to deal with an increasing prevalence of multimorbidity in an ageing population, we need to know about disease combinations so we can design best practice guidelines for clinicians [22]. In order to explore the combinations between systemic diseases, we created a network graph of the disease combinations, which facilitates a visual understanding of all combinations.

Our analysis of a large representative dataset showed that multimorbidity was common. Out of all inpatients who had two or more diseases 48.25% were identified as having multimorbidity; this proportion was much higher than in other studies [23,24]. This result might be explained by the fact that our subjects were inpatients, as different study populations likely played a key role in the discrepancy [25,26]. However, this study focused on only inpatients in general hospitals. Inpatients with multiple disorders will have poor functional capacity, poor quality of life and extended hospital stays, and they need a general service. The need for several healthcare providers to treat multiple diseases across different medical specialties makes it difficult to provide a generalist service. A strong, generalist primary care team is the most obvious way to provide continuity, coordination, and, above all, individualized care for people with multimorbidity to minimize future disability and morbidity.

Furthermore, we found that the prevalence of multimorbidity was much higher in males than in females; this result might be ascribed to differences in body function, e.g., the life expectancy of females is 5 years longer than that of males [27]. Another possible explanation is the great differences in the habits and diets between genders; for example, males tend to consume alcohol, smoke and eat meat more often than females [28,29,30]. Further, endocrine, nutritional and metabolic diseases (ENM) and diseases of the circulatory system (CirS) had the highest weights in both males and females, but the “ENM–CirS” combination in males was relatively more prominent than that in females. It is suggested that the “ENM–CirS” combination be given more attention, especially in males. 

The other notable finding was that the prevalence of multimorbidity was higher in old people ((146,190+93,882+40,451+20,712)/557,614 = 54.02%) than in young or middle-aged people (45.98%). This result agrees with results of previous work that showed that there was a substantial association between age and multimorbidity and that the prevalence of multimorbidity tends to be increased in older adults [25,31,32]. A possible reason for this might be that body immunity and function are greater in young people than in old people, so the proportion of the number of diseases was lowest in those 0 to 17 years old. In addition, respiratory system diseases (ResS) had the highest weight in multimorbidity in the age group 0~17 years. This may be related to low respiratory immunity in children, large temperature differences in northeast China, and environmental pollution [33,34]. Thus, multimorbidity dominated by respiratory system diseases (ResS) should be the focus of attention given to young people.

The health challenges faced by adults are more complex than those faced by young people, as body immunity and function decline with age; therefore, research on adult multimorbidity cannot be overlooked. The proportion of the number of diseases increased in those 18 to 64 years old, and ENM (endocrine, nutritional and metabolic diseases) and CirS (circulatory system diseases) had higher weight than other systemic diseases in multimorbidity. Although those 18 to 64 years old had a denser multimorbidity network, the “ResS-CirS” combination in the age group 85 years or older was more marked than other combinations, where their CirS (circulatory system diseases) occupied a large percentage compared with that of those 18 to 64 years old. These findings suggest that different key prevention methods should be targeted towards different age groups.

Jilin Province has a temperate continental monsoon climate, and we speculate that inhabitants consume high amounts of animal fat and salt and few vegetables [35]. Additionally, the population at middle age is under substantial mental stress, which leads to people feeling run-down and ill. Notably, the proportion of three or more diseases was the highest in those aged 55 and older. Geriatricians play a significant role in the provision of care for the frailest elderly patients with multimorbidity, but in most countries, these patients are treated in primary care facilities regardless of the critical connections between different diseases.

Finally, health insurance schemes are a microcosm of different professions. However, we found that the multimorbidity networks of four kinds of health insurance schemes (excluding others) were similar, and the “CirS-ENM” combination was more marked than other combinations in the multimorbidity networks. This also indirectly explains the existence of certain internal multimorbidity correlations, which will not substantially change on the basis of gender, age and occupation.

Some limitations should be noted here. On the one hand, only gender, age and health insurance scheme were investigated in the study, but other factors that might have effects on multimorbidity were worthy of further study. On the other hand, the participants in the study were inpatients, which might cause bias. Underlying these limitations is the lack of an internationally accepted standard for measuring multimorbidity. A clear conceptual framework must be developed that includes a consistent approach to the measurement of multimorbidity and age categorization in the study population to allow for comparison between studies and populations.

## 5. Conclusions

Different strategies should be separately developed to prevent multimorbidity in different age groups. Multimorbidity with respiratory system diseases in young people should not be overlooked. Additionally, effective prevention efforts that target endocrine, nutritional and metabolic diseases and circulatory system diseases are needed in middle aged and old people. Finally, knowledge of the common combinations of multimorbidity may help in planning the health services needed in the future, and the healthcare system requires further modifications to accurately identify and effectively manage patients with multimorbidity.

## Figures and Tables

**Table 1:** Descriptive characteristics of the patients by the number of diseases.

	n (%)	1	2	≥3	*χ* ^2^	*P*
All patients	1,155,734 (100%)	598,120 (51.75%)	354,787 (30.70%)	202,827 (17.55%)		
Gender						
Male	608,943 (52.69%)	297,967 (48.93%)	196,980 (32.35%)	113,996 (18.72%)	4125.02	<0.001
Female	546,791 (47.31%)	300,153 (54.89%)	157,807 (28.86%)	88,831 (16.25%)		
Age (years)						
0~	55,198 (4.78%)	37,385 (67.73%)	13,519 (24.49%)	4294 (7.78%)	32,416.92	<0.001
18~	116,520 (10.08%)	80,048 (68.70%)	26,167 (22.46%)	10,305 (8.84%)		
45~	420,757 (36.41%)	218,663 (51.97%)	128,460 (30.53%)	73,634 (17.50%)		
65~	466,980 (40.41%)	226,908 (48.59%)	146,190 (31.31%)	93,882 (20.10%)		
85~	96,279 (8.33%)	35,116 (36.47%)	40,451 (42.01%)	20,712 (21.51%)		
Health insurance schemes						
UEBMI	521,337 (45.11%)	259,243 (49.73%)	157,185 (30.15%)	104,909 (20.12%)	24,816.04	<0.001
URBMI	248,988 (21.54%)	119,751 (48.10%)	82,401 (33.09%)	46,836 (18.81%)		
NRCMS	197,144 (17.06%)	116,834 (59.26%)	54,593 (27.69%)	25,717 (13.04%)		
USP	151,632 (13.12%)	92,223 (60.82%)	44,127 (29.10%)	15,282 (10.08%)		
Others *	36,633 (3.17%)	10,069 (27.49%)	16,481 (44.99%)	10,083 (27.52%)		

UEBMI: Urban Employee Basic Medical Insurance; URBMI: Urban Resident Basic Medical Insurance; NRCMS: New Rural Cooperative Medical Scheme; USP: uninsured patients; * “others” included commercial health insurance, work-related injury insurance, maternity and medical assistance.

**Table 2:** The Abbreviation of the 13 systemic diseases.

The Name of the Systemic Diseases	Abbreviation
Neoplasms	Neo
Diseases of the blood and blood-forming organs and certain disorders involving the immune mechanism	BBI
Endocrine, nutritional and metabolic diseases	ENM
Mental and behavioral disorders	MB
Diseases of the nervous system	NerS
Diseases of the eye and adnexa	EA
Diseases of the ear and mastoid process	EM
Diseases of the circulatory system	CirS
Diseases of the respiratory system	ResS
Diseases of the digestive system	DigS
Diseases of the skin and subcutaneous tissue	SkSt
Diseases of the musculoskeletal system and connective tissue	MuSC
Diseases of the genitourinary system	GenS

**Table 3:** The proportions of the ten most common two-disease combinations by gender.

	Men	Women
1	ENM-CirS (18.50%)	ENM-CirS (16.73%)
2	CirS-ResS (13.37%)	CirS-ResS (9.08%)
3	ENM-ResS (6.06%)	CirS-GenS (5.87%)
4	NerS-CirS (5.22%)	CirS-DigS (5.28%)
5	CirS-DigS (5.05%)	ENM-ResS (5.07%)
6	ENM-NerS (4.77%)	NerS-CirS (4.46%)
7	ENM-DigS (4.68%)	ENM-DigS (4.41%)
8	CirS-GenS (4.29%)	ENM-GenS (4.23%)
9	ENM-GenS (3.87%)	BBI-GenS (4.22%)
10	ResS-DigS (3.35%)	ENM-NerS (3.90%)

## Supplementary Materials

The following are available online at https://www.mdpi.com/1660-4601/17/6/1817/s1. Table S1: International Statistical Classification of Diseases and Related Health Problems 10th Revision; Table S2: The proportions of ten most common two-disease combinations by age in males; Table S3: The proportions of ten most common two-disease combinations by age in females; Table S4: The proportions of ten most common two-disease combinations by health insurance scheme.
