# Supplementary material for: Multimorbidity Analysis of 13 Systemic Diseases in Northeast China
Source: Int J Environ Res Public Health. 2020 Mar 11;17(6):1817. doi: 10.3390/ijerph17061817 (PMC7142480; doi:10.3390/ijerph17061817)
Supplement: Supplementary file 1 [file ijerph-17-01817-s001.pdf]

## Supplementary Materials

# Multimorbidity Analysis of 13 systemic diseases in Northeast China

**Jianxing Yu [1], Fangying Song [1], Yingying Li [1], Zhou Zheng [1], Huanhuan Jia [1], Yuzhe Sun [1], Lina Jin [2] and Xihe Yu [1,*]**

[1] Social Medicine and Health Service Management, School of Public Health, No. 1163 Xinmin Street, Jilin University, Changchun, Jilin, China,130021.

[2] Epidemiology and Biostatistics, School of Public Health, NO. 1163 Xinmin Street, Jilin University, Changchun, Jilin, China, 130021.

* Correspondence and requests for materials should be addressed to Xihe Yu (email: xhyu@jlu.edu.cn)

**1. Choosing 13 systemic diseases**

According to the International Statistical Classification of Diseases and Related Health Problems 10th Revision (ICD), the diseases and related health problem were divided into 21 categories (See details in Table 1S). Besides, NO.I, NO.XV~XXI were excluded becasue these diseases were infectious disease, congenital diseases, injury, poisoning, pregnancy, childbirth and the puerperium and others. Thus, we finally choose the 13 systemic diseases (NO. II~ XIV) in this study.

**Table S1.** International Statistical Classification of Diseases and Related Health Problems 10th Revision.

| NO. | International Statistical Classification of Diseases and Related Health Problems 10th Revision |
|---|---|
| I | Certain infectious and parasitic diseases |
| II | Neoplasms |
| III | Diseases of the blood and blood-forming organs and certain disorders involving the immune mechanism |
| IV | Endocrine, nutritional and metabolic diseases |
| V | Mental and behavioural disorders |
| VI | Diseases of the nervous system |
| VII | Diseases of the eye and adnexa |
| VIII | Diseases of the ear and mastoid process |
| IX | Diseases of the circulatory system |
| X | Diseases of the respiratory system |
| XI | Diseases of the digestive system |
| XII | Diseases of the skin and subcutaneous tissue |
| XIII | Diseases of the musculoskeletal system and connective tissue |
| XIV | Diseases of the genitourinary system |
| XV | Pregnancy, childbirth and the puerperium |
| XVI | Certain conditions originating in the perinatal period |
| XVII | Congenital malformations, deformations and chromosomal abnormalities |
| XVIII | Symptoms, signs and abnormal clinical and laboratory findings, not elsewhere classified |

| | |
|---|---|
| XIX | Injury, poisoning and certain other consequences of external causes |
| XX | External causes of morbidity and mortality |
| XXI | Factors influencing health status and contact with health services |
| XXII | Codes for special purposes |

## 2. Ten most common two-disease combinations

**Table S2.** The proportions of ten most common two-disease combinations by age in males

| | 0~ | 18~ | 45~ | 65~ | 85~ |
|---|---|---|---|---|---|
| 1 | CirS-ResS(27.61%) | CirS-ENM(12.85%) | CirS-ENM(22.22%) | CirS-ENM(18.99%) | ResS-CirS(33.51%) |
| 2 | ResS-DigS(8.42%) | DigS-ENM(12.44%) | NerS-ENM(7.52%) | ResS-CirS(15.24%) | CirS-ENM(12.01%) |
| 3 | BBI-ResS(8.39%) | NerS-ENM(6.03%) | CirS-NerS(7.22%) | ResS-ENM(8.04%) | ResS-ENM(10.02%) |
| 4 | CirS-DigS(6.48%) | GenS-ENM(5.74%) | DigS-ENM(6.85%) | NerS-CirS(4.90%) | DigS-ResS(6.30%) |
| 5 | ResS-EM(4.26%) | DigS-CirS(5.67%) | DigS-CirS(5.64%) | CirS-GenS(4.83%) | GenS-CirS(6.21%) |
| 6 | CirS-ENM(3.66%) | GenS-CirS(4.74%) | CirS-ResS(5.30%) | DigS-CirS(3.98%) | DigS-CirS(5.94%) |
| 7 | ResS-ENM(3.64%) | DigS-ResS(3.74%) | GenS-ENM(4.04%) | ENM-GenS(3.70%) | GenS-ENM(3.74%) |
| 8 | CirS-BBI(3.22%) | GenS-BBI(3.60%) | Neo-DigS(3.76%) | NerS-ENM(3.54%) | GenS-ResS(3.50%) |
| 9 | DigS-ENM(2.93%) | ResS-ENM(3.60%) | ResS-ENM(3.24%) | DigS-Neo(3.44%) | CirS-NerS(2.15%) |
| 10 | ResS-NerS(2.91%) | CirS-NerS(3.12%) | GenS-CirS(3.22%) | Neo-CirS(3.39%) | CirS-BBI(1.86%) |

**Table S3.** The proportions of ten most common two-disease combinations by age in females

| | 0~ | 18~ | 45~ | 65~ | 85~ |
|---|---|---|---|---|---|
| 1 | CirS-ResS(26.16%) | DigS-ENM(8.08%) | CirS-ENM(16.58%) | CirS-ENM(19.00%) | ResS-CirS(21.39%) |
| 2 | ResS-DigS(8.27%) | GenS-Neo(7.97%) | CirS-NerS(5.63%) | ResS-CirS(10.19%) | CirS-ENM(15.36%) |
| 3 | ResS-BBI(7.70%) | GenS-BBI(7.63%) | NerS-ENM(5.55%) | GenS-CirS(7.29%) | ResS-ENM(11.66%) |
| 4 | DigS-CirS(6.96%) | ENM-Neo(6.30%) | DigS-ENM(5.15%) | DigS-CirS(5.65%) | DigS-CirS(9.96%) |
| 5 | CirS-ENM(4.17%) | ResS-ENM(5.35%) | GenS-ENM(4.68%) | ResS-ENM(5.11%) | GenS-CirS(4.81%) |
| 6 | ResS-EM(3.64%) | GenS-ENM(4.95%) | GenS-CirS(4.67%) | GenS-BBI(4.73%) | DigS-Neo(3.62%) |
| 7 | ResS-ENM(3.50%) | CirS-ENM(4.57%) | ResS-CirS(4.60%) | CirS-NerS(4.59%) | GenS-ResS(3.09%) |
| 8 | DigS-ENM(3.08%) | BBI-Neo(4.30%) | DigS-CirS(4.14%) | GenS-ENM(4.43%) | DigS-ResS(2.68%) |
| 9 | GenS-ResS(2.94%) | DigS-Neo(4.11%) | DigS-Neo(3.78%) | DigS-ENM(3.95%) | CirS-BBI(2.45%) |
| 10 | ResS-NerS(2.76%) | GenS-DigS(3.64%) | ResS-ENM(3.63%) | CirS-BBI(3.71%) | CirS-NerS(2.14%) |

**Table S4.** The proportions of ten most common two-disease combinations by health insurance scheme

| | UEBMI | URBMI | NRCMS | USP | Others* |
|---|---|---|---|---|---|
| 1 | CirS-ENM(19.44%) | CirS-ENM(18.83%) | CirS-ENM(14.24%) | ResS-CirS(16.08%) | ResS-CirS(33.52%) |
| 2 | ResS-CirS(8.56%) | ResS-CirS(11.27%) | ResS-CirS(11.62%) | CirS-ENM(13.40%) | CirS-ENM(13.07%) |
| 3 | CirS-NerS(6.13%) | ResS-ENM(6.02%) | ResS-ENM(6.32%) | ResS-ENM(5.91%) | ResS-ENM(9.76%) |
| 4 | NerS-ENM(6.06%) | GenS-CirS(5.85%) | GenS-CirS(5.11%) | DigS-CirS(5.19%) | DigS-CirS(9.47%) |

| | | | | | |
|---|---|---|---|---|---|
| 5 | DigS-CirS(4.91%) | DigS-ENM(5.20%) | DigS-CirS(4.57%) | DigS-ENM(4.37%) | DigS-ResS(8.56%) |
| 6 | GenS-CirS(4.88%) | DigS-CirS(5.13%) | GenS-BBI(4.32%) | GenS-CirS(3.72%) | GenS-CirS(4.01%) |
| 7 | ResS-ENM(4.78%) | CirS-NerS(4.55%) | DigS-Neo(4.14%) | DigS-Neo(3.50%) | GenS-ENM(3.51%) |
| 8 | DigS-ENM(4.76%) | GenS-ENM(4.33%) | GenS-ENM(4.13%) | CirS-NerS(3.42%) | GenS-ResS(2.56%) |
| 9 | GenS-ENM(4.05%) | NerS-ENM(4.07%) | DigS-ENM(3.70%) | GenS-ENM(3.28%) | ResS-Neo(2.42%) |
| 10 | DigS-Neo(2.95%) | GenS-BBI(3.93%) | CirS-BBI(3.63%) | DigS-ResS(3.03%) | DigS-ENM(2.12%) |

UEBMI ：Urban Employee Basic Medical Insurance; URBMI: Urban Resident Basic Medical Insurance; NRCMS: New Rural Cooperative Medical Scheme; USP: uninsured patients; *"others" included commercial health insurance, work-related injury insurance, maternity, medical assistance.
